# Sequel and Fatal Termination of a Case of Lymphatic Enlargement of the Right Leg and Thigh

**Published:** 1878-12

**Authors:** W. H. Day

**Affiliations:** London


### Original Communications.

### THE SEQUEL AND FATAL TERMINATION OF A CASE OF LYMPHATIC ENLARGEMENT OF THE RIGHT LEG AND THIGH.

By W. H. Day, M. D., London.

(*A paper read to the London Pathological Society, May* 24, 1878.)

[Through the kindness of the President of the London Pathological Society, we were recently put into communication with the author of the subjoined paper, which we desired to consult. Dr. Day has kindly forwarded to us advance sheets of the Transactions of the London Pathological Society, from which we reproduce his interesting narrative.—Ed.]

In order that this remarkable case may be understood by members of the Society who are unacquainted with the history and report as described in the Society's Transactions (Vol. II., p. 104), I deem it advisable to briefly recapitulate the leading symptoms as recorded there for the information of members who were not present at the reading of the paper.

Nine years ago I recorded the case of T. C. D., at that time 7 years of age. The right leg was noticed to be larger than the left when he was 2½ years old, causing no inconvenience, and not affecting the general health. When four years old the swelling extended into the thigh, and like the leg was firm and inelastic; yellowish white spots appeared over the head of the tibia, looking like small encysted growths, containing cheesy matter, and subsequently drying up and disappearing. After exercise, fatigue or an attack of indigestion, the leg became hard, brawny, and covered with patches of erythema. When the inflammatory blush disappeared, the limb presented yellowish patches like pityriasis versicolor. In 1866 he could not flex the limb, and the symptoms were thought attributable to obstruction of nearly the whole length of the femoral vein. The prepuce became much hypertrophied, and, in 1867, necessitated removal from the constant dribbling of urine. In 1868 a pearly vesicle like herpes formed on the edge of the prepuce, which ruptured and discharged a chylous fluid like lymph. After this the limb lessened in size. In January, 1869, as much as half a pint of creamy fluid escaped from this small orifice, and the following day there was shivering and rigor; smaller and transparent vesicles began to form on the glans penis, discharging lymph from time to time, and he was very ill. The toes were swelled and of waxy whiteness, whilst the diseased limb, in circumference, was three inches larger than the left. For the next three years he was tolerably well, but suffered at uncertain intervals from attacks of weakness, and pain and swelling in the limb.

September 15, 1872.—After two days of fatigue and running about, he woke up with pain and stiffness in the leg, the foot and toes being more particularly attacked. When lying down he could not raise the limb, and this loss of power accompanied each attack. There were thirst, headache, and pungent heat of skin; temperature 39°, pulse 130 full, with considerable tension. Calomel 0.06 and a saline aperient draught emptied the bowels and relieved the pain and excitement. On the 17th he was easier, and all the discomfort was in the foot. On the 18th (next day) pain came on in the knee-joint, which was exquisitely tender and sensitive; he cried out from the suffering, and could get no ease at night. A dose of chloral failed to procure him any sleep. He remained in considerable pain till the 20th, when I was summoned to him in the country. The knee was partially flexed on the thigh—it was hot, swollen and pale; there was effusion beneath the integuments on either side of the patella, but apparently not in the joint itself. A warm lead lotion was kept constantly applied under oiled silk, and the swelling diminished. The urine was densely loaded with pink lithates, and on standing in a two-ounce bottle only four grams were clear and free from sediment. Citrate of potash in water was given as a drink. When the leg was at rest he remained free from suffering, and was able to travel from Cambridgeshire to London on the 23d, but the slightest movement of the joint, or the least pressure, gave pain.

The ten days' confinement to bed had greatly reduced his strength, and his loss of flesh was very noticeable ; he was pallid too and exsanguine, and his voice too at these times became husky and weak.

24th.—There was no pain in the knee except on pressure and the movement of the limb ; the swelling was especially marked over the tubercle of the tibia, and occupied the site of the two old dried lymphatics ; there was distinct fluctuation, but no redness or throbbing. The reduction in the size of the thigh had never been so marked, and the skin, instead of being stretched and hard, was lax and supple.

The following were the measurements of the respective limbs :—

| DISEASED OR RIGHT LIMB. | | | SOUND LIMB. | | |
|---|---|---|---|---|---|
| Above knee | 35.0 | centim. | Above knee | 25.0 | centim. |
| Middle of thigh | 37.5 | " | Middle of thigh | 31.5 | " |
| Upper third do. | 39.0 | " | Upper third do. | 32.5 | " |
| Below knee | 30.0 | " | Below knee | 30.0 | " |
| Calf | 32.5 | " | Calf | 20.0 | " |
| Above ankle | 22.5 | " | Above ankle | 17.5 | " |

27th.—The swelling of the knee was subsiding—great tenderness over the tibia previously alluded to.

October 2d.—For some months he had been greatly troubled with nettle-rash, the back and shoulders were mostly affected, but the diseased limb was not more involved than the rest of the body. He experienced a good deal of pain in getting down stairs. The chief symptoms at this time were debility, pallor of face, loss of appetite, and inability to straighten or bear any weight on the affected limb.

8th.—For the last four days he had complained of great pain in the *left* inguinal region, and there was an enlarged inguinal gland the size of a small nut ; to this spot he referred his suffering, and any movement of the limb increased it ; he was exhausted and feverish towards the evening, and in the night perspired a good deal. His appetite was very capricious, and he was fretful and irritable on slight provocation. The affected limb had a very numbed sensation, which probably arose from exhaustion and weakness of the muscular system. Any attempt to move the limb threw the muscles into spasmodic action, and he would shiver like a person in the cold stage of ague.

22d.—There had been increasing pain in the right knee-joint for a week. He often awoke in the night from the pain, and no ease could be obtained till he had taken a full sedative. I had my doubts whether mischief was not proceeding in the joint, but Sir James Paget, who saw him, considered the pain neuralgic, and advised a continuance of quinine, brandy, and nourishment.

November 5th.—There had been no ease since last report, and the suffering was pitiable to witness, coming on in paroxysms at night, sometimes lasting for hours, and preventing sleep. Sir J. Paget saw him again, and advised bromide of potassium, a liniment of aconite, chloroform and opium, which seemed for a time to relieve him. About the close of the month the pain in the knee-joint departed.

December 6th.— For a week he had complained of pain in the right hip, below and to the right of the anterior superior spinous process of the ilium. Yesterday, and again to-day, there had been great pain coming on in the three outer toes of the right side, and a general aching of the limb. When pain was present the three toes trembled like the rest of the limb, and were pale, smooth and shining. There was no oscillation of temperature, which was normal, morning and evening scarcely varying; the night perspiration had subsided, but the pulse was weak and unsteady, and his appetite failed, perhaps by reason of the sedative, which he was compelled to take at night.

18th.—He came down to dine to-day, but not without pain. For the last week he had suffered from extreme nausea, and at times severe vomiting and retching. The pain had come on in a moment when most occupied with his amusements. Of late he has looked very sallow, and complained of pain over the back of his neck, and his eyes have become weak and inflamed. I now prescribed steel wine and arsenic twice a day after meals.

25th.—He came down stairs to dine, and was tolerably well, though he looked dreadfully pale and exhausted; two days later there was agony in the leg for hours, and he was very sick throughout the day without apparent cause.

1873, January 8th.—Having been free from pain for a week, he went out for a drive. As medicines failed to afford relief they were given up.

March 1st.—He passed a round worm, having for weeks suffered from nausea and vomiting.

19th.—Was kept awake with agony in the knee and starting of the limb; he had no rest till he had taken a third dose of chlorodyne.

October 18th.—For the last month he had been free from pain, sleeping well and enjoying his food, but easily tired after any kind of exertion. At Felixstowe, in Suffolk, where he stayed for six weeks, he did not get on so well, the place being cold and the comforts much less than at home. No discharge of lymph since March—a period of six months.

Used crutches for 1½ years, and during this time was comparatively free from pain.

1875.—Sufficiently well to attend with tolerable regularity at school; he had comparative freedom from pain, which was now always in the foot, and there was an occasional discharge from the foot also.

1876.—In July (weather very hot), after much fatigue, he was seized with a severe feverish attack, lasting four days, during which the leg was very hot and red; he was much weakened, and after going from home, took cold, from which he suffered for two months, accompanied with cough. During this time the discharge occurred frequently, and he was much reduced and prostrate.

In October, when at the seaside, and after running about, he had a sharp febrile attack; on the 28th, when he attempted to get out of bed, he fell; the big leg, which had been stiff throughout the night, proving useless to him and painful. A week's rest in bed restored him, though, on first getting up, he was obliged to use his crutches again for a few days.

On December 13th, he took cold and had a return of cough, with slight bronchitis, and kept his bed and room for a month, getting out again on January 12th, 1877.

All the autumn term his tutor had come to him at home; but at his especial wish, after Christmas he resumed his school life, and for the next month enjoyed unusually good health, practicing the organ daily, and working six hours. He suffered but little pain, and had only a slight occasional discharge from the foot.

On January 29th, 1877, whilst sitting at dinner, his mother noticed that the lower half of his face turned livid, and the servants noticed it also, but he did not complain. On the following day he returned from school with a headache, which had come on an hour previously. He went to bed directly, and remained there part of next day. He was unusually well the three following days, till taken ill in church.

February 4th.—Went to church well at 11 a.m., and whilst in an erect posture, suddenly fell down in a state of syncope at 12. On rallying, he was led out into the open air, when he complained of great pain over the hypogastrium, and he was deadly pale. He, however, was able to walk home with assistance, and just as he entered his bed-room he began to shiver, and was placed on the bed in a state of collapse, with imperceptible pulse, livid hands and features; the tremor was as great as was seen in the cold stage of ague. He referred all his pain to the hypogastrium. Brandy was given at once, and t. opii 1 C. C. thrown up the rectum.

12:45.—The pain had now shifted into the leg and knee, and, as it was very severe, the sedative injection was repeated.

1 p. m., free vomiting took place.

1:30, vomited a quantity of bilious fluid.

2:30, reaction fully established; temp. 39.5°; pulse 136 full and bounding; skin hot and pungent; intense thirst; urine passed freely, copious and clear.

4:15, restless, in great pain, and a draught of hydrate of chloral and bromide of potassium, of each one gram, was taken.

5:0, in great agony and begs for relief. About 8 grams of bichloride of mythelene were inhaled, extending over half an hour.

7:0, free from pain at intervals for a few minutes together; skin moist; temp. 39.1°; pulse 132; R. 56, short and catching.

11:30, temp. 40.2°; pulse 144; R. 48; urine passed; conjuntivæ injected; much wandering. A soap-and-water enema brought away some scybalæ from the bowel and a little fæcal fluid.

5th.—1:15 a. m., temp. 40.3°; pulse 140; R. 44, very shallow; sleeping.

2:15, temp. 40.5°; pulse 140; R. 48; sleeping restlessly; drank iced water, but did not understand anything addressed to him.

3:15, aspect wild, staring and excited; has tetanic spasms in the arms, and both hands spasmodically clenched; *iced water cap applied* to the head.

4:0, he looks brighter, swallows well, and the hands are relaxed; temp. 40.6°; pulse 144; R. 40.

5:0, temp. 40.6°; pulse 140; R. 44.

6:0, temp. 40.3°.

7:0, temp. 39.9°; pulse 142; R. 44.

8:30, bowels acted.

9:30, bowels acted.

10:0, temp. 39.5°.

10:30, very copious action of bowels.

11:45, temp. 39.4°; pulse 140, very weak and small; face cool and lips rather dusky; breathing quiet and regular, but still quick; intellect quite clear. Drank a small quantity of beef-tea and iced champagne.

1:30 p. m., temp. 39.2°; pulse 140, weak; face alternately flushes and turns pale; is very restless and unconscious, and the iced cap is kept on with difficulty.

2:30, temp. 39.2°; pulse 140, very weak; R. 44; the bowels acted unconsciously, after which he asked for some cold water; face ashy.

3:30, bowels again acted; sick after a little champagne and beef-tea.

4:10, sickness and pain in stomach; slight motion; cannot rest quiet for a moment together; t. opii 1 C. C. per rectum.

6:0, temp. 40.0° ; pulse 160, fluttering; R. 48; rested after the enema, and the bowels have not since acted. Two decigrams of quinine, t. opii 1 C. C. and beef-tea gm. 60 ordered to be thrown into the rectum every two hours.

7:0, temp. 40.0° ; pulse 160; R. 56; unconscious.

9:0, temp. 39.9° ; pulse 160, so small that it could scarcely be recognized; R. 56; unconsciousness continues; extremities feel rather cold, and cheeks also; enemata returned.

12:0, temp. 39.8°.

6.—2:30 a. m., temp. 39.8° ; R. 36, irregular.

3:0, pulseless; heart's action feeble and tumultuous, but the sounds could be distinctly recognized; constantly tossing about, so that it was not easy to ascertain whether there was a cardiac bruit; the aspect and general symptoms seem to indicate the formation of clot in both cavities.

4:30, temp. 39.6° ; R. 44, very irregular and shallow—expiration much prolonged; still pulseless, and unable to swallow.

6:30, too restless to have the temperature taken.

9:0, pulsation can be felt faintly in both brachials, but not below; lividity increases, but no stertor; pupils small.

11:0, temp. 39.1°.

12:30, about half a pint of high-colored urine drawn off, sp. gr. 1014, acid, faint cloud on heating, and not quite clear by nitric acid. He grew gradually worse, tossing to and fro, throwing up his hands, and rolling his head from side to side; his hands became very cold and livid, and at 3 p. m. his breathing was more labored and less rapid; the expiration at one time was very long, but about 3:30 it changed its character, whilst the inspiration became long and labored, both acts being free from stertor. About this time his face grew paler and colder, and he expired at 3:45 p. m.

The post-mortem was conducted with great care by Dr. Wickham Legg and Dr. J. Ormerod nineteen hours after death.

The following measurements were taken: from the left anterior superior iliac spine to top of left patella 44 Cm.; from right anterior superior spine to top of right patella 42 Cm. From left anterior superior iliac spine to left inner malleolus 83

Cm.; from right anterior superior iliac spine to right inner malleolus 88 Cm.

| | | |
|---|---|---|
| Left limb, half way between knee and ankle | 25.6 | centim. |
| Right limb | 39.4 | " |
| Largest circumference of left knee | 30.6 | " |
| " " " right knee | 44.4 | " |
| Girth of left thigh at a point half way between anterior superior iliac spine and patella | 38.0 | " |
| Do. right do | 51.0 | " |

Nothing noteworthy was found about the heart. The lungs were œdematous, but no other morbid change was present. All the viscera of the belly were natural.

There was no obstruction or old thrombus found in the vena cava or iliac veins. The lumbar plexus on both sides was natural to the naked eye. There was no increase in size of the inguinal glands. The large size of the right leg seemed due to an increase in the subcutaneous tissues solely, not to the muscles. No other appearance of disease could be perceived.

Dr. Gowers examined the spinal cord with the microscope and found it quite natural.

*Remarks.*—The results of the post-mortem examination explain little or nothing, on account of our possessing insufficient data of the physiology, and still feebler knowledge of the pathology of the lymphatic system. There was no evidence from this examination of any obstruction to the femoral vein, and apparently no enlargement of the arterial or venous trunks, though it seemed highly probable that some great disturbance took place from time to time owing to the heat of the limb, pain and swelling, and especially those rapid local changes in the limb which ensued shortly after the fatal seizure. It may be said that the last attack was merely the severe form of many previous, but less formidable attacks. At no time was the urine chylous.

This case, in the character of the discharge, and large size of the limb, resembles those described by Mr. Sidney Jones ("Path. Trans." vol. xxvi., 1875), Mr. Berkeley Hill, and Dr. Cholmeley ("Clin. Trans." 1869), but it differs from these, and all I have been able to meet with, in the increased length and growth of the bones of the affected limb. In the report furnished by Mr. Callender and Dr. Burdon-Sanderson, they say that " a perfectly similar case is recorded by Dujardin in 1854." Even in Mr. Thomas Smith's case of nævoid elephantiasis (" St. Barth. Hospl. Rep." vol. v.), where the enlarged limb was three inches longer than the sound leg, " the lengthening was due to the excessive hypertrophy of the integuments over the sole and heel."

This case would be placed by the systematic surgeon under the category of " lymphatic elephantiasis," Mr. T. Smith (" Nævoid Elephantiasis," " St. Barthol. Hospl. Rep." vol. v., 1869) having already distinguished three varieties of elephantiasis arabum —lymphatic, vascular, and climatic—the last being the swollen leg seen in tropical climates. Indeed, referring to my first report of this case, he has himself already reckoned it as an instance of the lymphatic variety of elephantiasis.

